# Crystal structures and Hirshfeld surface analysis of 5-amino-1-(4-meth­oxy­phen­yl)pyrazole-4-carb­ox­ylic acid and 5-amino-3-(4-meth­oxy­phen­yl)isoxazole

**DOI:** 10.1107/S2056989022001827

**Published:** 2022-02-25

**Authors:** Chris J. Pintro, Analeece K. Long, Allison J. Amonette, James M. Lobue, Clifford W. Padgett

**Affiliations:** a Georgia Southern University, 11935 Abercorn St., Department of Chemistry and, Biochemistry, Savannah GA 30458, USA

**Keywords:** X-ray diffraction, crystal structure, isoxazole, Hirshfeld surface

## Abstract

The title compounds, C_11_H_11_N_3_O_3_, (I), and C_10_H_10_N_2_O_2_, (II), are commercially available and were crystallized from ethyl acetate solution. The dihedral angle between the pyrazole and phenyl rings in (I) is 52.34 (7)° and the equivalent angle between the isoxazole and phenyl rings in (II) is 7.30 (13)°. In the crystal of (I), the mol­ecules form carb­oxy­lic acid inversion dimers with an *R*(8) ring motif *via* pairwise O—H⋯O hydrogen bonds. In the crystal of (II), the mol­ecules are linked *via* N—H⋯N hydrogen bonds forming chains propagating along [010] with a *C*(5) motif. A weak N—H⋯π inter­action also features in the packing of (II). Hirshfeld surface analysis was used to explore the inter­molecular contacts in the crystals of both title compounds: the most important contacts for (I) are H⋯H (41.5%) and O⋯H/H⋯O (22.4%). For (II), the most significant contact percentages are H⋯H (36.1%) followed by C⋯H/H⋯C (31.3%).

## Abstract 2

The title compounds feature O—H⋯O carb­oxy­lic-acid inversion dimers and N—H⋯N chains in their extended structures.

## Chemical context

This report is one of a series on the structures and hydrogen-bonding motifs in small-mol­ecule aromatic amino carb­oxy­lic acids (I) and small-mol­ecule aromatic amino compounds (II). This study follows other reports including, for example, 3-amino­pyrazine-2-carb­oxy­lic acid (Dobson & Gerkin, 1996 ▸), 5-amino­isophthalic acid hemihydrate (Dobson & Gerkin, 1998 ▸), and 1,4-di­benzyl­piperazine-2,5-dione (Nunez, *et al.*, 2004 ▸). We now describe the structures of 5-amino-1-(4-meth­oxy­phen­yl)-pyrazole-4-carb­oxy­lic acid, (I) and 5-amino-3-(4-meth­oxy­phen­yl)isoxazole, (II). 
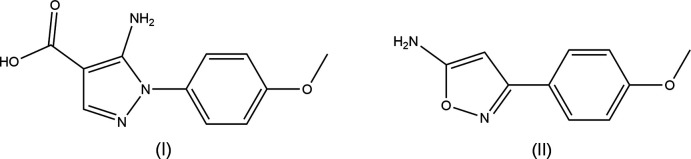


## Structural commentary

**Figure 1:**
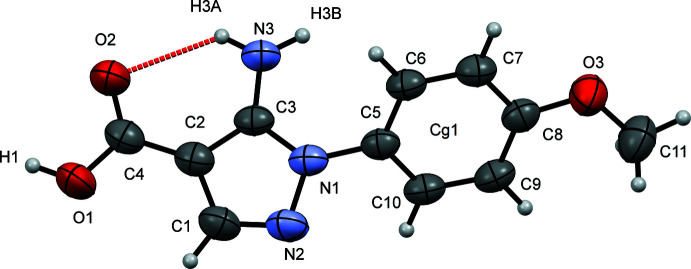
The mol­ecular structure of (I) with displacement ellipsoids drawn at the 50% probability level. The intra­molecular hydrogen bond is represented by a red dashed line.

The mol­ecular structure of compound (I) is shown in Fig. 1 ▸. The pyrazole ring (r.m.s. deviation = 0.010 Å) is rotated by 52.34 (7)° relative to the phenyl ring (r.m.s. deviation = 0.010 Å), which is the primary contribution to the general non-planarity of the mol­ecule. An intra­molecular N3—H3*A*⋯O2 hydrogen bond is observed (Table 1 ▸ and Fig. 1 ▸). This bond forms an *S*(6) ring motif (Fig. 1 ▸ and Table 1 ▸) with an N3⋯O2 distance of 2.941 (3) Å. This is a common feature in analogous compounds (such as those listed in the *Database survey*). The C3—N3 distance of 1.353 (2) Å is typical for an amino group bound to an aromatic ring. The carb­oxy­lic carbon–oxygen distances are 1.255 (2) and 1.316 (2) for C4—O2 and C4—O1, respectively, indicating that the former bond may be affected by the intra­molecular N—H⋯O hydrogen bond.

**Figure 2:**
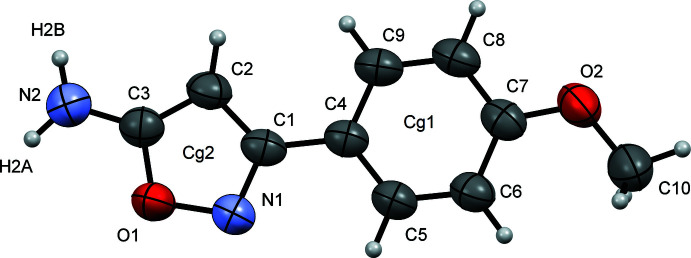
The mol­ecular structure of (II) with displacement ellipsoids drawn at the 50% probability level.

The mol­ecular structure of compound (II) is shown in Fig. 2 ▸. The angle between the phenyl and isoxazole rings is 7.30 (13)°, resulting in the overall mol­ecule being close to planar with the r.m.s. deviation of all non-hydrogen atoms being 0.054 Å. The N1—O1 distance is 1.434 (4) Å and is consistent with other isoxazoles (see *Database survey* section). The C3—N2 distance is 1.350 (5) Å and is typical of an amino group bound to an aromatic ring.

## Supra­molecular features

**Figure 3:**
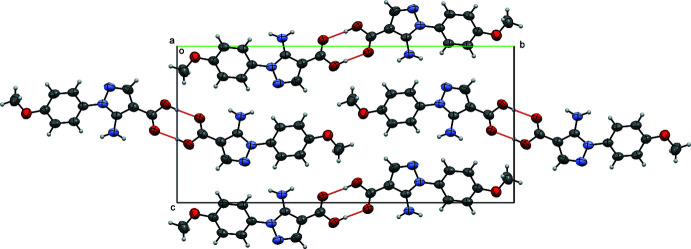
A view along the *a*-axis direction of the crystal packing of (I) with hydrogen bonds shown as red dashed lines.

In the extended structure of (I), the mol­ecules form centrosymmetric hydrogen-bonded dimers *via* the O1—H1⋯O2^i^ [symmetry code: (i) −*x* + 1, −*y*, −*z* + 1] link to generate an *R*(8) loop with O⋯O = 2.649 (2) Å, see Table 1 ▸ and Fig. 3 ▸. These dimers are linked *via* π–π inter­actions, notably weak stacking inter­actions between the 4-meth­oxy­phenyl rings [*Cg*1⋯*Cg*1 (*x* + 1, *y*, *z*) = 3.9608 (4) Å, where *Cg*1 is the centroid of the C5–C10 ring] along the *a*-axis direction.

**Figure 4:**
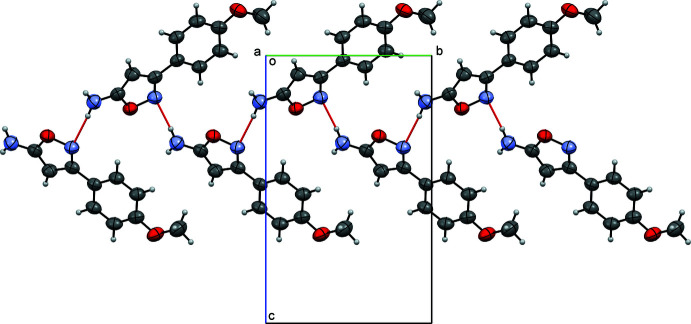
A view along the *a*-axis direction of the crystal packing of (II) with hydrogen bonds shown as red dashed lines.

In the packing of (II), the mol­ecules form hydrogen-bonded chains running along the *b*-axis direction *via* the N2—H2*A*⋯N1^i^ hydrogen bond [symmetry code: (i) −*x* + 1, *y* + 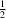 , −*z* + 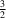 ] hydrogen bond forms a *C*(5) chain motif with an N⋯N distance of 3.003 (5) Å, see Table 2 ▸ and Fig. 4 ▸. No π–π inter­actions are observed.

## Hirshfeld surface analysis

The inter­molecular inter­actions were further investigated by qu­anti­tative analysis of the Hirshfeld surface, and visualized with *Crystal Explorer 17.5* (Turner *et al.*, 2017 ▸; Spackman *et al.*, 2009 ▸) and the two-dimensional fingerprint plots (McKinnon *et al.*, 2007 ▸). The shorter and longer contacts are indicated as red and blue spots, respectively, on the Hirshfeld surfaces, and contacts with distances approximately equal to the sum of the van der Waals radii are colored white. The function *d*_norm_ is a ratio enclosing the distances of any surface point to the nearest inter­ior (*d*_i_) and exterior (*d*_e_) atom and the van der Waals (vdW) radii of the atoms. The *d*_norm_ plots were mapped with a color scale between −0.18 au (blue) and +1.4 au (red).

**Figure 5:**
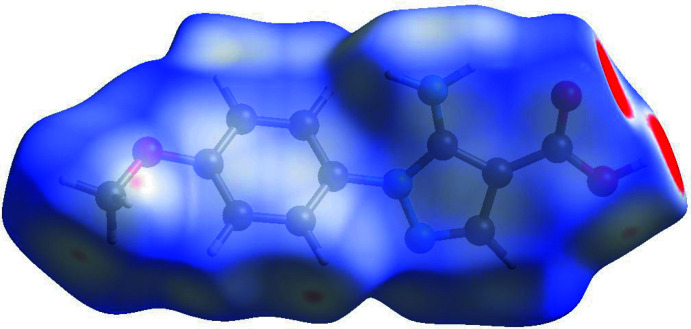
Hirshfeld surface for (I) mapped over *d*_norm_.

Fig. 5 ▸. shows the *d*_norm_ surface of compound (I). The most intense red spots on the *d*_norm_ surface correspond to the O1—H1⋯O2 inter­actions. The red and blue triangles on the shape-index surface indicate that there are weak π-stacking inter­actions in the crystal structure. Analysis of the two-dimensional fingerprint plots indicate that the H⋯H (41.5%) inter­actions are the major factor in the crystal packing with O⋯H/H⋯O (22.4%) inter­actions making the next highest contribution. The percentage contributions of other significant contacts are: C⋯H/H⋯C (13.1%) and N⋯H/ H⋯N (8.7%).

**Figure 6:**
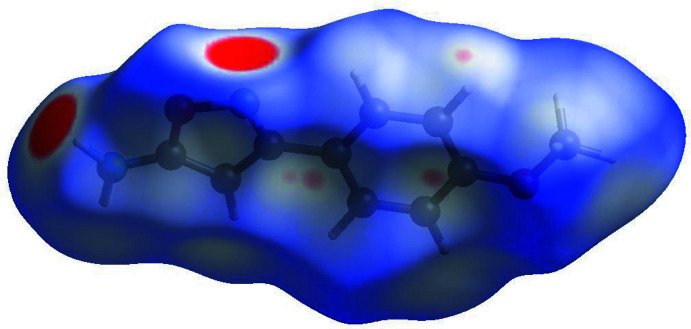
Hirshfeld surface for (II) mapped over *d*_norm_.

Fig. 6 ▸ shows the *d*_norm_ surface of compound (II). The large red spots represent N2—H2*A*⋯N1 inter­actions. Some additional inter­actions indicated by very light-red spots correspond to contacts around phenyl ring and isoxazole rings: N2—H2*B*⋯*Cg*1^ii^ [2.97 (4) Å], C6—H6⋯*Cg*1^iii^ (2.86 Å) and C9—H9⋯*Cg*2^ii^ (2.86 Å) [symmetry codes: (ii) *x* + 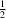 , −*y* + 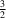 , −*z* + 1; (iii) *x* − 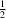 , −*y* + 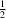 , −*z* + 1; *Cg*1 and *Cg*2 are the centroids of the O1/N1/C1–C3 and C4–C9 rings, respectively]. Analysis of the two-dimensional fingerprint plots indicates that the H⋯H (36.1%) inter­actions are the major factor in the crystal packing with C⋯H/H⋯C (31.3%) contacts making the next highest contribution. The percentage contributions of other weak inter­actions are: O⋯H/H⋯O (17.3%) and N⋯H/ H⋯N (12.1%). Figures showing the shape-index surface for each compound and the overall fingerprint plots are included in the supporting information.

## Database survey

A search of the Cambridge Structural Database (CSD, version 5.42, update of November 2020; Groom *et al.*, 2016 ▸) gave 13 hits for the 3-phenyl­isoxazol-5-amine moiety. The four most closely related compounds are: 5-di­acetyl­amino-3,4-di­phenyl­isoxazole (CSD refcode ACPIXZ; Simon *et al.*, 1974 ▸), 1,5-dimethyl-4-phenyl-3-(3-phenyl-1,2-oxazol-5-yl)imidazol­id­in-2-one (HOGYAE; Li *et al.*, 2007 ▸), *N-*4-dimethyl-*N*-[3-{4-(tri­fluoro­meth­yl)phen­yl]-1,2-oxazol-5-yl}benzene-1-sulfonamide (XOSHUL; Chen & Cui, 2019 ▸), 3-phenyl-5-(1*H*-pyrazol-1-yl)-1,2-oxazole (ZEVGIT; Mikhailov *et al.*, 2018 ▸).

A similar search gave 14 hits for the 5-amino-1-phenyl-1*H*-pyrazole-4-carb­oxy­lic acid moiety. The seven most closely related compounds are: ethyl 1-(4-chloro-2-nitro­phen­yl)-5-nitro-4,5-di­hydro-1*H*-pyrazole-4-carboxyl­ate (GOLHEV; Zia-ur-Rehman *et al.*, 2009 ▸), 5-amino-1-phenyl-3-(tri­fluoro­meth­yl)-1*H*-pyrazole-4-carb­oxy­lic acid (HUDDEQ; Caruso *et al.*, 2009 ▸), 5-amino-1-phenyl-1*H*-pyrazole-4-carb­oxy­lic acid (KODXIL; Zia-ur-Rehman *et al.*, 2008 ▸), ethyl 5-amino-1-(2,4-di­nitro­phen­yl)-1*H*-pyrazole-4-carboxyl­ate (QAHJER; Ghorab *et al.*, 2016 ▸), ethyl 5-amino-1-phenyl-1*H*-pyrazole-4-carboxyl­ate (RUVHUO, Soares *et al.*, 2020 ▸), ethyl 5-amino-1-(4-sulfamoylphen­yl)-1*H*-pyrazole-4-carboxyl­ate (XUTZIX; Ibrahim *et al.*, 2015 ▸) and 2-eth­oxy­ethyl 5-amino-1-(2,4-di­methyl­phen­yl)-3-(methyl­thio)-1*H*-pyrazole-4-carboxyl­ate (YOYHOK, Liu *et al.*, 2009 ▸).

## Synthesis and crystallization

Compounds (I) and (II) are commercially available and were purchased from Aldrich. Both were dissolved in ethyl acetate until saturated and these solutions were allowed to evaporate slowly at room temperature, which resulted in X-ray quality crystals.

## Refinement

Crystal data, data collection, and structure refinement details are summarized in Table 3 ▸. All carbon-bound H atoms were positioned geometrically and refined as riding, with C—H = 0.95 or 0.98 Å and *U*_iso_(H) = 1.2*U*_eq_(C) or 1.5*U*_eq_(methyl C). In order to ensure a chemically meaningful O—H distance in (I), this was restrained to a target value of 0.84 (2) Å and *U*_iso_(H) = 1.5*U*_eq_(O). In (I), the amino H atoms were located in a difference-Fourier map. In (II), the N—H distances were restrained to a target value of 0.84 (2) Å and *U*_iso_(H) = 1.5*U*_eq_(N). The absolute structure of (II) was indeterminate based on the present refinement.

## Supplementary Material

Crystal structure: contains datablock(s) I, II. DOI: 10.1107/S2056989022001827/hb8013sup1.cif

Structure factors: contains datablock(s) I. DOI: 10.1107/S2056989022001827/hb8013Isup2.hkl

Structure factors: contains datablock(s) II. DOI: 10.1107/S2056989022001827/hb8013IIsup3.hkl

Supporting information file. DOI: 10.1107/S2056989022001827/hb8013Isup4.cml

Supporting information file. DOI: 10.1107/S2056989022001827/hb8013IIsup5.cml

Shape-index and fingerprint plots for compounds (I) and (II). DOI: 10.1107/S2056989022001827/hb8013sup6.pdf

CCDC references: 2152583, 2152582

Additional supporting information:  crystallographic information; 3D view; checkCIF report

## Figures and Tables

**Table 1:** Hydrogen-bond geometry (Å, °) for (I)

*D*—H⋯*A*	*D*—H	H⋯*A*	*D*⋯*A*	*D*—H⋯*A*
N3—H3*A*⋯O2	0.87 (2)	2.32 (2)	2.941 (3)	128.5 (18)
O1—H1⋯O2^i^	0.90 (2)	1.75 (2)	2.649 (2)	176 (3)

Symmetry code: (i) 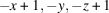 .

**Table 2:** Hydrogen-bond geometry (Å, °) for (II) *Cg*2 is the centroid of the C4–C9 ring.

*D*—H⋯*A*	*D*—H	H⋯*A*	*D*⋯*A*	*D*—H⋯*A*
N2—H2*A*⋯N1^i^	0.89 (3)	2.12 (3)	3.003 (5)	174 (6)
N2—H2*B*⋯*Cg*2^ii^	0.85 (2)	2.97 (4)	3.709 (4)	147 (4)

Symmetry codes: (i) 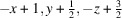 ; (ii) 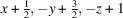 .

**Table 3:** Experimental details

	(I)	(II)
Crystal data		
Chemical formula	C_11_H_11_N_3_O_3_	C_10_H_10_N_2_O_2_
*M*_r_	233.23	190.20
Crystal system, space group	Monoclinic, *P*2_1_/*n*	Orthorhombic, *P*2_1_2_1_2_1_
Temperature (K)	170	170
*a*, *b*, *c* (Å)	3.9608 (4), 24.104 (3), 11.1762 (10)	7.6496 (11), 8.7565 (15), 14.128 (2)
α, β, γ (°)	90, 90.189 (9), 90	90, 90, 90
*V* (Å^3^)	1067.0 (2)	946.4 (3)
*Z*	4	4
Radiation type	Mo *K*α	Mo *K*α
μ (mm^−1^)	0.11	0.10
Crystal size (mm)	0.5 × 0.2 × 0.2	0.4 × 0.2 × 0.2

Data collection		
Diffractometer	Rigaku XtaLAB mini	Rigaku XtaLAB mini
Absorption correction	Multi-scan (*CrysAlis PRO*; Rigaku OD, 2018 ▸)	Multi-scan (*CrysAlis PRO*; Rigaku OD, 2018 ▸)
*T*_min_, *T*_max_	0.975, 1.000	0.757, 1.000
No. of measured, independent and observed [*I* > 2σ(*I*)] reflections	8175, 2937, 1667	6912, 2635, 1344
*R*_int_	0.032	0.050
(sin θ/λ)_max_ (Å^−1^)	0.694	0.694

Refinement		
*R*[*F* ^2^ > 2σ(*F* ^2^)], *wR*(*F* ^2^), *S*	0.049, 0.155, 1.03	0.052, 0.168, 1.02
No. of reflections	2937	2635
No. of parameters	168	137
No. of restraints	1	2
H-atom treatment	H atoms treated by a mixture of independent and constrained refinement	H atoms treated by a mixture of independent and constrained refinement
Δρ_max_, Δρ_min_ (e Å^−3^)	0.16, −0.19	0.17, −0.16
Absolute structure	–	Flack *x* determined using 385 quotients [(*I* ^+^)−(*I* ^−^)]/[(*I* ^+^)+(*I* ^−^)] (Parsons *et al.*, 2013 ▸)
Absolute structure parameter	–	−0.7 (10)

Computer programs: *CrysAlis PRO* (Rigaku OD, 2018 ▸), *SHELXT2014/5* (Sheldrick, 2015*a* ▸), *SHELXL2018/1* (Sheldrick, 2015*b* ▸), and *OLEX2* (Dolomanov *et al.*, 2009 ▸).

## supplementary crystallographic information

### 5-Amino-1-(4-methoxyphenyl)pyrazole-4-carboxylic acid (I) Crystal data

**Table:**

C_11_H_11_N_3_O_3_	*F*(000) = 488
*M**_r_* = 233.23	*D*_x_ = 1.452 Mg m^−^^3^
Monoclinic, *P*2_1_/*n*	Mo *K*α radiation, λ = 0.71073 Å
*a* = 3.9608 (4) Å	Cell parameters from 1271 reflections
*b* = 24.104 (3) Å	θ = 2.0–23.7°
*c* = 11.1762 (10) Å	µ = 0.11 mm^−^^1^
β = 90.189 (9)°	*T* = 170 K
*V* = 1067.0 (2) Å^3^	Block, clear colourless
*Z* = 4	0.5 × 0.2 × 0.2 mm

### 5-Amino-1-(4-methoxyphenyl)pyrazole-4-carboxylic acid (I) Data collection

**Table:**

Rigaku XtaLAB mini diffractometer	2937 independent reflections
Radiation source: Sealed Tube, Rigaku (Mo) X-ray Source	1667 reflections with *I* > 2σ(*I*)
Graphite Monochromator monochromator	*R*_int_ = 0.032
Detector resolution: 13.6612 pixels mm^-1^	θ_max_ = 29.6°, θ_min_ = 2.0°
profile data from ω–scans	*h* = −5→5
Absorption correction: multi-scan (CrysalisPro; Rigaku OD, 2018)	*k* = −33→33
*T*_min_ = 0.975, *T*_max_ = 1.000	*l* = −15→14
8175 measured reflections	

### 5-Amino-1-(4-methoxyphenyl)pyrazole-4-carboxylic acid (I) Refinement

**Table:**

Refinement on *F*^2^	Hydrogen site location: mixed
Least-squares matrix: full	H atoms treated by a mixture of independent and constrained refinement
*R*[*F*^2^ > 2σ(*F*^2^)] = 0.049	*w* = 1/[σ^2^(*F*_o_^2^) + (0.060*P*)^2^ + 0.1763*P*] where *P* = (*F*_o_^2^ + 2*F*_c_^2^)/3
*wR*(*F*^2^) = 0.155	(Δ/σ)_max_ < 0.001
*S* = 1.03	Δρ_max_ = 0.16 e Å^−^^3^
2937 reflections	Δρ_min_ = −0.19 e Å^−^^3^
168 parameters	Extinction correction: SHELXL2018/1 (Sheldrick, 2015b), Fc^*^=kFc[1+0.001xFc^2^λ^3^/sin(2θ)]^-1/4^
1 restraint	Extinction coefficient: 0.017 (3)
Primary atom site location: dual	

### 5-Amino-1-(4-methoxyphenyl)pyrazole-4-carboxylic acid (I) Special details

**Table:**

Geometry. All esds (except the esd in the dihedral angle between two l.s. planes) are estimated using the full covariance matrix. The cell esds are taken into account individually in the estimation of esds in distances, angles and torsion angles; correlations between esds in cell parameters are only used when they are defined by crystal symmetry. An approximate (isotropic) treatment of cell esds is used for estimating esds involving l.s. planes.

### 5-Amino-1-(4-methoxyphenyl)pyrazole-4-carboxylic acid (I) Fractional atomic coordinates and isotropic or equivalent isotropic displacement parameters (Å^2^)

**Table:**

	*x*	*y*	*z*	*U*_iso_*/*U*_eq_	
O1	0.3397 (4)	0.03421 (7)	0.62727 (13)	0.0697 (5)	
H1	0.370 (8)	0.0005 (8)	0.594 (2)	0.113 (11)*	
C1	0.2875 (5)	0.14586 (10)	0.72321 (18)	0.0595 (5)	
H1A	0.208584	0.120332	0.781520	0.071*	
N1	0.4218 (4)	0.22042 (7)	0.63223 (12)	0.0495 (4)	
C2	0.4182 (5)	0.12959 (8)	0.61090 (17)	0.0519 (5)	
O2	0.5820 (4)	0.06706 (6)	0.46040 (12)	0.0648 (4)	
N2	0.2864 (5)	0.19977 (8)	0.73857 (14)	0.0608 (5)	
O3	0.5775 (4)	0.44729 (6)	0.57003 (13)	0.0699 (5)	
C3	0.5066 (4)	0.17936 (8)	0.55617 (15)	0.0463 (4)	
N3	0.6542 (5)	0.18839 (9)	0.44896 (14)	0.0573 (5)	
H3A	0.704 (5)	0.1581 (9)	0.4099 (19)	0.060 (6)*	
H3B	0.692 (6)	0.2227 (10)	0.422 (2)	0.070 (7)*	
C4	0.4535 (5)	0.07533 (9)	0.56133 (17)	0.0545 (5)	
C5	0.4620 (4)	0.27870 (8)	0.61733 (15)	0.0462 (4)	
C6	0.3325 (5)	0.30549 (8)	0.51751 (15)	0.0495 (5)	
H6	0.215830	0.285018	0.457648	0.059*	
C7	0.3738 (5)	0.36188 (9)	0.50557 (16)	0.0522 (5)	
H7	0.285859	0.380302	0.437091	0.063*	
C8	0.5434 (5)	0.39210 (8)	0.59310 (16)	0.0504 (5)	
C9	0.6654 (5)	0.36518 (8)	0.69428 (16)	0.0524 (5)	
H9	0.774143	0.385704	0.755892	0.063*	
C10	0.6278 (5)	0.30858 (8)	0.70480 (15)	0.0505 (5)	
H10	0.716871	0.289972	0.772872	0.061*	
C11	0.7499 (6)	0.48080 (10)	0.6567 (2)	0.0770 (7)	
H11A	0.979212	0.466515	0.668620	0.115*	
H11B	0.627391	0.479613	0.732705	0.115*	
H11C	0.761020	0.519180	0.628081	0.115*	

### 5-Amino-1-(4-methoxyphenyl)pyrazole-4-carboxylic acid (I) Atomic displacement parameters (Å^2^)

**Table:**

	*U*^11^	*U*^22^	*U*^33^	*U*^12^	*U*^13^	*U*^23^
O1	0.0905 (12)	0.0604 (10)	0.0584 (9)	−0.0021 (9)	0.0143 (8)	0.0150 (7)
C1	0.0614 (12)	0.0677 (14)	0.0495 (11)	−0.0015 (10)	0.0083 (9)	0.0107 (9)
N1	0.0505 (9)	0.0608 (10)	0.0373 (8)	−0.0023 (7)	0.0036 (7)	0.0019 (6)
C2	0.0491 (11)	0.0609 (12)	0.0457 (10)	−0.0016 (9)	−0.0010 (8)	0.0053 (8)
O2	0.0815 (11)	0.0609 (9)	0.0521 (8)	−0.0018 (7)	0.0098 (7)	0.0048 (6)
N2	0.0691 (11)	0.0708 (12)	0.0427 (8)	−0.0016 (9)	0.0140 (8)	0.0069 (7)
O3	0.0846 (11)	0.0573 (9)	0.0678 (9)	−0.0025 (8)	−0.0131 (8)	−0.0037 (7)
C3	0.0403 (9)	0.0623 (12)	0.0362 (8)	−0.0017 (8)	−0.0026 (7)	0.0013 (8)
N3	0.0731 (12)	0.0585 (11)	0.0402 (9)	−0.0022 (9)	0.0104 (8)	0.0009 (8)
C4	0.0528 (11)	0.0610 (13)	0.0495 (11)	0.0002 (9)	−0.0029 (9)	0.0104 (9)
C5	0.0404 (9)	0.0593 (11)	0.0390 (9)	−0.0021 (8)	0.0038 (7)	−0.0005 (7)
C6	0.0468 (10)	0.0638 (12)	0.0379 (9)	−0.0034 (9)	−0.0023 (8)	−0.0026 (8)
C7	0.0511 (11)	0.0647 (13)	0.0409 (9)	0.0033 (9)	−0.0025 (8)	0.0011 (8)
C8	0.0480 (10)	0.0576 (12)	0.0455 (10)	0.0026 (8)	0.0030 (8)	−0.0053 (8)
C9	0.0495 (11)	0.0664 (13)	0.0413 (9)	0.0001 (9)	−0.0026 (8)	−0.0091 (8)
C10	0.0483 (10)	0.0669 (13)	0.0364 (9)	0.0025 (9)	−0.0014 (8)	−0.0024 (8)
C11	0.0820 (16)	0.0637 (14)	0.0852 (16)	−0.0024 (12)	−0.0122 (13)	−0.0173 (12)

### 5-Amino-1-(4-methoxyphenyl)pyrazole-4-carboxylic acid (I) Geometric parameters (Å, º)

**Table:**

O1—H1	0.901 (17)	N3—H3B	0.90 (2)
O1—C4	1.316 (2)	C5—C6	1.386 (2)
C1—H1A	0.9500	C5—C10	1.379 (2)
C1—C2	1.415 (3)	C6—H6	0.9500
C1—N2	1.311 (3)	C6—C7	1.376 (3)
N1—N2	1.397 (2)	C7—H7	0.9500
N1—C3	1.348 (2)	C7—C8	1.391 (3)
N1—C5	1.424 (2)	C8—C9	1.389 (3)
C2—C3	1.392 (3)	C9—H9	0.9500
C2—C4	1.427 (3)	C9—C10	1.378 (3)
O2—C4	1.255 (2)	C10—H10	0.9500
O3—C8	1.362 (2)	C11—H11A	0.9800
O3—C11	1.433 (3)	C11—H11B	0.9800
C3—N3	1.353 (2)	C11—H11C	0.9800
N3—H3A	0.87 (2)		
			
C4—O1—H1	113.6 (19)	C10—C5—C6	120.09 (19)
C2—C1—H1A	123.4	C5—C6—H6	120.2
N2—C1—H1A	123.4	C7—C6—C5	119.65 (17)
N2—C1—C2	113.11 (18)	C7—C6—H6	120.2
N2—N1—C5	119.63 (15)	C6—C7—H7	119.8
C3—N1—N2	111.84 (16)	C6—C7—C8	120.44 (18)
C3—N1—C5	128.51 (15)	C8—C7—H7	119.8
C1—C2—C4	129.42 (19)	O3—C8—C7	115.24 (17)
C3—C2—C1	104.13 (18)	O3—C8—C9	125.16 (17)
C3—C2—C4	126.45 (18)	C9—C8—C7	119.60 (19)
C1—N2—N1	103.90 (16)	C8—C9—H9	120.2
C8—O3—C11	118.03 (17)	C10—C9—C8	119.65 (17)
N1—C3—C2	106.99 (16)	C10—C9—H9	120.2
N1—C3—N3	123.35 (18)	C5—C10—H10	119.7
N3—C3—C2	129.65 (19)	C9—C10—C5	120.54 (17)
C3—N3—H3A	114.1 (14)	C9—C10—H10	119.7
C3—N3—H3B	121.7 (15)	O3—C11—H11A	109.5
H3A—N3—H3B	124 (2)	O3—C11—H11B	109.5
O1—C4—C2	116.02 (18)	O3—C11—H11C	109.5
O2—C4—O1	121.64 (19)	H11A—C11—H11B	109.5
O2—C4—C2	122.34 (18)	H11A—C11—H11C	109.5
C6—C5—N1	120.85 (16)	H11B—C11—H11C	109.5
C10—C5—N1	119.05 (16)		
			
C1—C2—C3—N1	−1.5 (2)	C3—C2—C4—O1	−177.21 (18)
C1—C2—C3—N3	177.56 (19)	C3—C2—C4—O2	2.1 (3)
C1—C2—C4—O1	2.3 (3)	C4—C2—C3—N1	178.11 (18)
C1—C2—C4—O2	−178.46 (19)	C4—C2—C3—N3	−2.9 (3)
N1—C5—C6—C7	179.75 (16)	C5—N1—N2—C1	−179.45 (17)
N1—C5—C10—C9	−178.75 (16)	C5—N1—C3—C2	179.87 (16)
C2—C1—N2—N1	0.0 (2)	C5—N1—C3—N3	0.8 (3)
N2—C1—C2—C3	0.9 (2)	C5—C6—C7—C8	−0.2 (3)
N2—C1—C2—C4	−178.6 (2)	C6—C5—C10—C9	0.3 (3)
N2—N1—C3—C2	1.6 (2)	C6—C7—C8—O3	178.16 (17)
N2—N1—C3—N3	−177.50 (17)	C6—C7—C8—C9	−1.3 (3)
N2—N1—C5—C6	−127.58 (18)	C7—C8—C9—C10	2.3 (3)
N2—N1—C5—C10	51.4 (2)	C8—C9—C10—C5	−1.8 (3)
O3—C8—C9—C10	−177.11 (18)	C10—C5—C6—C7	0.8 (3)
C3—N1—N2—C1	−1.0 (2)	C11—O3—C8—C7	180.00 (19)
C3—N1—C5—C6	54.3 (3)	C11—O3—C8—C9	−0.5 (3)
C3—N1—C5—C10	−126.71 (19)		

### 5-Amino-1-(4-methoxyphenyl)pyrazole-4-carboxylic acid (I) Hydrogen-bond geometry (Å, º)

**Table:**

*D*—H···*A*	*D*—H	H···*A*	*D*···*A*	*D*—H···*A*
N3—H3*A*···O2	0.87 (2)	2.32 (2)	2.941 (3)	128.5 (18)
O1—H1···O2^i^	0.90 (2)	1.75 (2)	2.649 (2)	176 (3)

Symmetry code: (i) −*x*+1, −*y*, −*z*+1.

### 5-Amino-3-(4-methoxyphenyl)isoxazole (II) Crystal data

**Table:**

C_10_H_10_N_2_O_2_	*D*_x_ = 1.335 Mg m^−^^3^
*M**_r_* = 190.20	Mo *K*α radiation, λ = 0.71073 Å
Orthorhombic, *P*2_1_2_1_2_1_	Cell parameters from 1405 reflections
*a* = 7.6496 (11) Å	θ = 2.7–22.5°
*b* = 8.7565 (15) Å	µ = 0.10 mm^−^^1^
*c* = 14.128 (2) Å	*T* = 170 K
*V* = 946.4 (3) Å^3^	Block, clear colourless
*Z* = 4	0.4 × 0.2 × 0.2 mm
*F*(000) = 400	

### 5-Amino-3-(4-methoxyphenyl)isoxazole (II) Data collection

**Table:**

Rigaku XtaLAB mini diffractometer	2635 independent reflections
Radiation source: fine-focus sealed X-ray tube, Rigaku (Mo) X-ray Source	1344 reflections with *I* > 2σ(*I*)
Graphite Monochromator monochromator	*R*_int_ = 0.050
Detector resolution: 13.6612 pixels mm^-1^	θ_max_ = 29.6°, θ_min_ = 2.7°
profile data from ω–scans	*h* = −10→9
Absorption correction: multi-scan (CrysalisPro; Rigaku OD, 2018)	*k* = −11→11
*T*_min_ = 0.757, *T*_max_ = 1.000	*l* = −11→19
6912 measured reflections	

### 5-Amino-3-(4-methoxyphenyl)isoxazole (II) Refinement

**Table:**

Refinement on *F*^2^	H atoms treated by a mixture of independent and constrained refinement
Least-squares matrix: full	*w* = 1/[σ^2^(*F*_o_^2^) + (0.0606*P*)^2^] where *P* = (*F*_o_^2^ + 2*F*_c_^2^)/3
*R*[*F*^2^ > 2σ(*F*^2^)] = 0.052	(Δ/σ)_max_ < 0.001
*wR*(*F*^2^) = 0.168	Δρ_max_ = 0.17 e Å^−^^3^
*S* = 1.02	Δρ_min_ = −0.16 e Å^−^^3^
2635 reflections	Extinction correction: SHELXL2018/1 (Sheldrick 2015b), Fc^*^=kFc[1+0.001xFc^2^λ^3^/sin(2θ)]^-1/4^
137 parameters	Extinction coefficient: 0.037 (7)
2 restraints	Absolute structure: Flack *x* determined using 385 quotients [(*I*^+^)-(*I*^-^)]/[(*I*^+^)+(*I*^-^)] (Parsons *et al.*, 2013)
Primary atom site location: dual	Absolute structure parameter: −0.7 (10)
Hydrogen site location: mixed	

### 5-Amino-3-(4-methoxyphenyl)isoxazole (II) Special details

**Table:**

Geometry. All esds (except the esd in the dihedral angle between two l.s. planes) are estimated using the full covariance matrix. The cell esds are taken into account individually in the estimation of esds in distances, angles and torsion angles; correlations between esds in cell parameters are only used when they are defined by crystal symmetry. An approximate (isotropic) treatment of cell esds is used for estimating esds involving l.s. planes.

### 5-Amino-3-(4-methoxyphenyl)isoxazole (II) Fractional atomic coordinates and isotropic or equivalent isotropic displacement parameters (Å^2^)

**Table:**

	*x*	*y*	*z*	*U*_iso_*/*U*_eq_	
O1	0.5261 (3)	0.8179 (3)	0.69875 (17)	0.0617 (7)	
C1	0.5966 (4)	0.6690 (4)	0.5813 (2)	0.0523 (8)	
N1	0.4969 (4)	0.6710 (4)	0.6566 (2)	0.0632 (8)	
O2	0.6029 (4)	0.1662 (3)	0.33196 (17)	0.0736 (8)	
N2	0.6808 (5)	1.0370 (5)	0.6730 (3)	0.0718 (10)	
C2	0.6906 (5)	0.8049 (4)	0.5699 (3)	0.0615 (10)	
H2	0.770803	0.829616	0.520934	0.074*	
C3	0.6421 (5)	0.8938 (4)	0.6445 (2)	0.0578 (9)	
C4	0.5977 (4)	0.5345 (4)	0.5186 (2)	0.0516 (8)	
C5	0.5115 (5)	0.4010 (4)	0.5422 (2)	0.0588 (9)	
H5	0.452653	0.395103	0.601305	0.071*	
C6	0.5081 (5)	0.2752 (5)	0.4821 (2)	0.0613 (10)	
H6	0.446575	0.185365	0.499673	0.074*	
C7	0.5963 (5)	0.2827 (5)	0.3959 (3)	0.0592 (10)	
C8	0.6848 (5)	0.4147 (5)	0.3719 (2)	0.0629 (10)	
H8	0.746554	0.419948	0.313670	0.076*	
C9	0.6843 (5)	0.5390 (5)	0.4321 (2)	0.0606 (10)	
H9	0.744260	0.629430	0.414044	0.073*	
C10	0.5103 (6)	0.0287 (5)	0.3529 (3)	0.0887 (14)	
H10A	0.533820	−0.047211	0.303518	0.133*	
H10B	0.384640	0.049924	0.355305	0.133*	
H10C	0.549038	−0.011138	0.414254	0.133*	
H2A	0.635 (9)	1.074 (6)	0.726 (3)	0.17 (3)*	
H2B	0.753 (5)	1.087 (5)	0.640 (3)	0.090 (17)*	

### 5-Amino-3-(4-methoxyphenyl)isoxazole (II) Atomic displacement parameters (Å^2^)

**Table:**

	*U*^11^	*U*^22^	*U*^33^	*U*^12^	*U*^13^	*U*^23^
O1	0.0673 (15)	0.0646 (17)	0.0531 (13)	−0.0011 (14)	0.0098 (11)	0.0035 (12)
C1	0.0433 (16)	0.065 (2)	0.0489 (17)	0.0065 (18)	0.0021 (14)	0.0079 (16)
N1	0.071 (2)	0.064 (2)	0.0551 (16)	−0.0038 (18)	0.0105 (15)	−0.0017 (15)
O2	0.0721 (17)	0.089 (2)	0.0600 (15)	−0.0106 (18)	0.0091 (13)	−0.0176 (14)
N2	0.080 (3)	0.065 (2)	0.071 (2)	−0.0054 (19)	0.0106 (19)	−0.0007 (18)
C2	0.056 (2)	0.070 (3)	0.058 (2)	−0.001 (2)	0.0137 (17)	0.0019 (19)
C3	0.056 (2)	0.063 (2)	0.0545 (19)	0.0013 (18)	−0.0014 (16)	0.0057 (18)
C4	0.0453 (17)	0.062 (2)	0.0477 (15)	0.0034 (17)	0.0009 (15)	0.0059 (15)
C5	0.057 (2)	0.070 (2)	0.0493 (18)	0.0015 (19)	0.0071 (16)	0.0024 (17)
C6	0.055 (2)	0.075 (2)	0.0539 (19)	−0.0026 (19)	0.0072 (18)	−0.0003 (18)
C7	0.0492 (18)	0.077 (3)	0.0512 (18)	0.0034 (19)	−0.0005 (17)	−0.0031 (17)
C8	0.055 (2)	0.083 (3)	0.0502 (19)	0.001 (2)	0.0097 (16)	0.0034 (19)
C9	0.056 (2)	0.073 (2)	0.0527 (19)	−0.001 (2)	0.0068 (16)	0.0096 (18)
C10	0.093 (3)	0.092 (3)	0.080 (3)	−0.019 (3)	0.015 (3)	−0.020 (3)

### 5-Amino-3-(4-methoxyphenyl)isoxazole (II) Geometric parameters (Å, º)

**Table:**

O1—N1	1.434 (4)	C4—C9	1.390 (5)
O1—C3	1.348 (4)	C5—H5	0.9500
C1—N1	1.310 (4)	C5—C6	1.391 (5)
C1—C2	1.399 (5)	C6—H6	0.9500
C1—C4	1.474 (5)	C6—C7	1.394 (5)
O2—C7	1.363 (5)	C7—C8	1.382 (6)
O2—C10	1.429 (5)	C8—H8	0.9500
N2—C3	1.350 (5)	C8—C9	1.381 (5)
N2—H2A	0.89 (3)	C9—H9	0.9500
N2—H2B	0.85 (2)	C10—H10A	0.9800
C2—H2	0.9500	C10—H10B	0.9800
C2—C3	1.362 (5)	C10—H10C	0.9800
C4—C5	1.383 (5)		
			
C3—O1—N1	108.0 (3)	C6—C5—H5	119.0
N1—C1—C2	112.4 (3)	C5—C6—H6	120.4
N1—C1—C4	120.2 (3)	C5—C6—C7	119.2 (4)
C2—C1—C4	127.4 (3)	C7—C6—H6	120.4
C1—N1—O1	105.0 (3)	O2—C7—C6	124.2 (4)
C7—O2—C10	118.4 (3)	O2—C7—C8	116.4 (3)
C3—N2—H2A	120 (4)	C8—C7—C6	119.3 (4)
C3—N2—H2B	117 (3)	C7—C8—H8	119.8
H2A—N2—H2B	122 (5)	C9—C8—C7	120.5 (3)
C1—C2—H2	127.5	C9—C8—H8	119.8
C3—C2—C1	104.9 (3)	C4—C9—H9	119.3
C3—C2—H2	127.5	C8—C9—C4	121.3 (4)
O1—C3—N2	115.7 (3)	C8—C9—H9	119.3
O1—C3—C2	109.7 (3)	O2—C10—H10A	109.5
N2—C3—C2	134.6 (4)	O2—C10—H10B	109.5
C5—C4—C1	121.8 (3)	O2—C10—H10C	109.5
C5—C4—C9	117.6 (3)	H10A—C10—H10B	109.5
C9—C4—C1	120.5 (3)	H10A—C10—H10C	109.5
C4—C5—H5	119.0	H10B—C10—H10C	109.5
C4—C5—C6	122.1 (3)		
			
C1—C2—C3—O1	0.2 (4)	C3—O1—N1—C1	0.2 (4)
C1—C2—C3—N2	−178.0 (4)	C4—C1—N1—O1	−178.9 (3)
C1—C4—C5—C6	178.2 (3)	C4—C1—C2—C3	178.6 (3)
C1—C4—C9—C8	−179.2 (3)	C4—C5—C6—C7	0.9 (6)
N1—O1—C3—N2	178.3 (3)	C5—C4—C9—C8	−0.2 (5)
N1—O1—C3—C2	−0.3 (4)	C5—C6—C7—O2	179.5 (4)
N1—C1—C2—C3	−0.1 (4)	C5—C6—C7—C8	0.0 (5)
N1—C1—C4—C5	−7.7 (5)	C6—C7—C8—C9	−0.9 (6)
N1—C1—C4—C9	171.3 (3)	C7—C8—C9—C4	1.0 (6)
O2—C7—C8—C9	179.5 (3)	C9—C4—C5—C6	−0.8 (5)
C2—C1—N1—O1	−0.1 (4)	C10—O2—C7—C6	1.7 (6)
C2—C1—C4—C5	173.7 (3)	C10—O2—C7—C8	−178.8 (4)
C2—C1—C4—C9	−7.3 (5)		

### 5-Amino-3-(4-methoxyphenyl)isoxazole (II) Hydrogen-bond geometry (Å, º)

*Cg*2 is the centroid of the C4–C9 ring.

**Table:**

*D*—H···*A*	*D*—H	H···*A*	*D*···*A*	*D*—H···*A*
N2—H2*A*···N1^i^	0.89 (3)	2.12 (3)	3.003 (5)	174 (6)
N2—H2*B*···*Cg*2^ii^	0.85 (2)	2.97 (4)	3.709 (4)	147 (4)

Symmetry codes: (i) −*x*+1, *y*+1/2, −*z*+3/2; (ii) *x*+1/2, −*y*+3/2, −*z*+1.

## References

[bb1] Caruso, F., Raimondi, M. V., Daidone, G., Pettinari, C. & Rossi, M. (2009). *Acta Cryst.* E**65**, o2173. 10.1107/S1600536809032188 PMC2970016 21577579

[bb2] Chen, C. & Cui, S. (2019). *J. Org. Chem.* **84**, 12157–12164. 10.1021/acs.joc.9b01430 31433177

[bb3] Dobson, A. J. & Gerkin, R. E. (1996). *Acta Cryst.* C**52**, 1512–1514. 10.1107/s0108270195016416 8766897

[bb4] Dobson, A. J. & Gerkin, R. E. (1998). *Acta Cryst.* C**54**, 1503–1505. 10.1107/s0108270198005915 9807799

[bb5] Dolomanov, O. V., Bourhis, L. J., Gildea, R. J., Howard, J. A. K. & Puschmann, H. (2009). *J. Appl. Cryst.* **42**, 339–341.

[bb6] Ghorab, M. M., Alsaid, M. S. & Ghabbour, H. A. (2016). *Z. Kristallogr. New Cryst. Struct.* **231**, 699–701.

[bb7] Groom, C. R., Bruno, I. J., Lightfoot, M. P. & Ward, S. C. (2016). *Acta Cryst.* B**72**, 171–179. 10.1107/S2052520616003954 PMC4822653 27048719

[bb8] Li, H., You, L., Zhang, X., Johnson, W. L., Figueroa, R. & Hsung, R. P. (2007). *Heterocycles*, **74**, 553–568.

[bb9] Ibrahim, H. S., Abou-Seri, S. M., Tanc, M., Elaasser, M. M., Abdel-Aziz, H. A. & Supuran, C. T. (2015). *Eur. J. Med. Chem.* **103**, 583–593. 10.1016/j.ejmech.2015.09.021 26408817

[bb10] Liu, Y., Liu, S., Li, Y., Song, H. & Wang, Q. (2009). *Bioorg. Med. Chem. Lett.* **19**, 2953–2956. 10.1016/j.bmcl.2009.04.048 19406637

[bb11] McKinnon, J. J., Jayatilaka, D. & Spackman, M. A. (2007). *Chem. Commun.* pp. 3814–3816. 10.1039/b704980c 18217656

[bb12] Mikhailov, K. I., Galenko, E. E., Galenko, A. V., Novikov, M. S., Ivanov, A. Y., Starova, G. L. & Khlebnikov, A. F. (2018). *J. Org. Chem.* **83**, 3177–3187. 10.1021/acs.joc.8b00069 29444569

[bb13] Nunez, L., Brown, J. D., Donnelly, A. M., Whitlock, C. R. & Dobson, A. J. (2004). *Acta Cryst.* E**60**, 2076–2078.

[bb14] Parsons, S., Flack, H. D. & Wagner, T. (2013). *Acta Cryst.* B**69**, 249–259. 10.1107/S2052519213010014 PMC3661305 23719469

[bb15] Rigaku OD (2018). *CrysAlis PRO*. Rigaku Oxford Diffraction, Yarnton, England.

[bb16] Sheldrick, G. M. (2015*a*). *Acta Cryst.* A**71**, 3–8.

[bb17] Sheldrick, G. M. (2015*b*). *Acta Cryst.* C**71**, 3–8.

[bb18] Simon, K., Sasvári, K., Dvortsák, P., Horváth, K. & Harsányi, K. (1974). *J. Chem. Soc. Perkin Trans. 2*, pp. 1409–1412.

[bb19] Soares, I. C., Junior, H. C. S., de Almeida, P. S. V. B., Alves, O. C., Soriano, S., Ferreira, G. F. & Guedes, G. P. (2020). *Inorg. Chem. Commun.* **121**, 108201.

[bb20] Spackman, M. A. & Jayatilaka, D. (2009). *CrystEngComm*, **11**, 19–32.

[bb21] Turner, M. J., McKinnon, J. J., Wolff, S. K., Grimwood, D. J., Spackman, P. R., Jayatilaka, D. & Spackman, M. A. (2017). *Crystal Explorer 17*. University of Western Australia. http://hirshfeldsurface.net.

[bb22] Zia-ur-Rehman, M., Elsegood, M. R. J., Akbar, N. & Shah Zaib Saleem, R. (2008). *Acta Cryst.* E**64**, o1312–o1313. 10.1107/S1600536808018394 PMC2961688 21202940

[bb23] Zia-ur-Rehman, M., Elsegood, M. R. J., Choudary, J. A., Fasih Ullah, M. & Siddiqui, H. L. (2009). *Acta Cryst.* E**65**, o275–o276. 10.1107/S1600536809000488 PMC2968147 21581889

