# Supplementary material for: Crystal structures and Hirshfeld surface analysis of 5-amino-1-(4-meth­oxy­phen­yl)pyrazole-4-carb­ox­ylic acid and 5-amino-3-(4-meth­oxy­phen­yl)isoxazole
Source: Acta Crystallogr E Crystallogr Commun. 2022 Feb 25;78(Pt 3):336–9. doi: 10.1107/S2056989022001827 (PMC8900502; doi:10.1107/S2056989022001827)
Supplement: Supplementary file 6 [file e-78-00336-sup6.pdf]

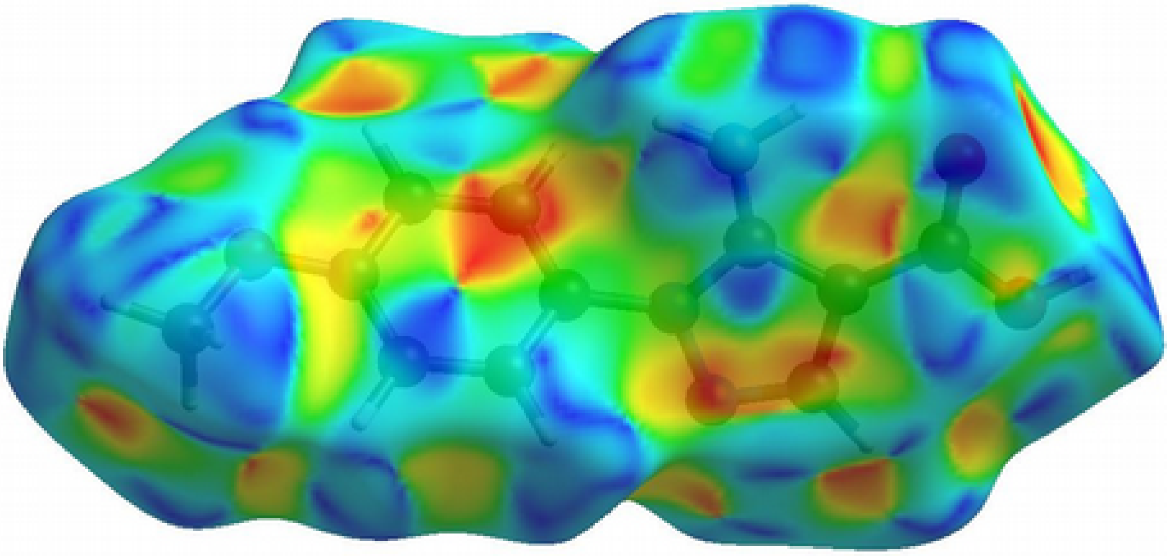

Hirshfeld surface of (I) plotted over shape-index.

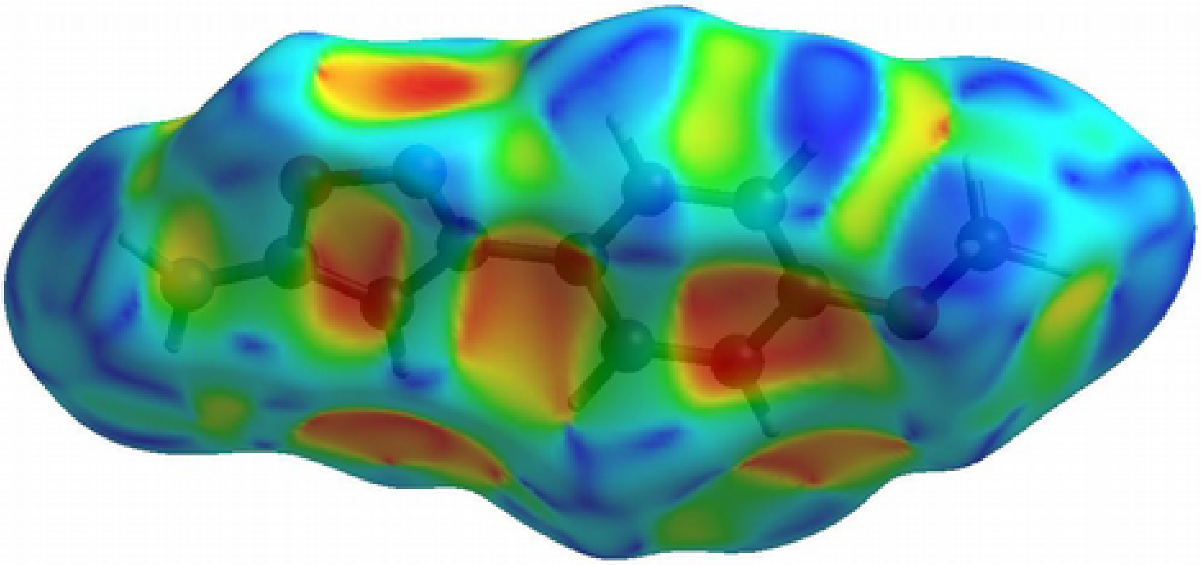

Hirshfeld surface of (II) plotted over shape-index.

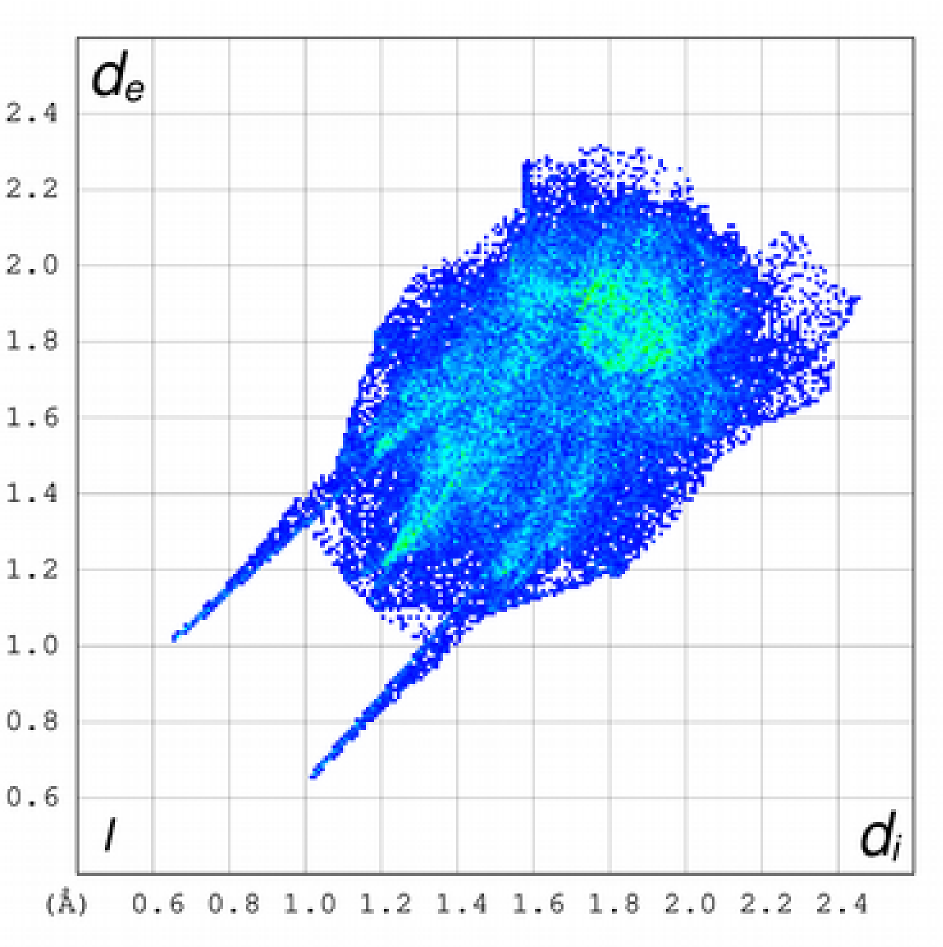

The full two-dimensional fingerprint plot for (I) showing all interactions.

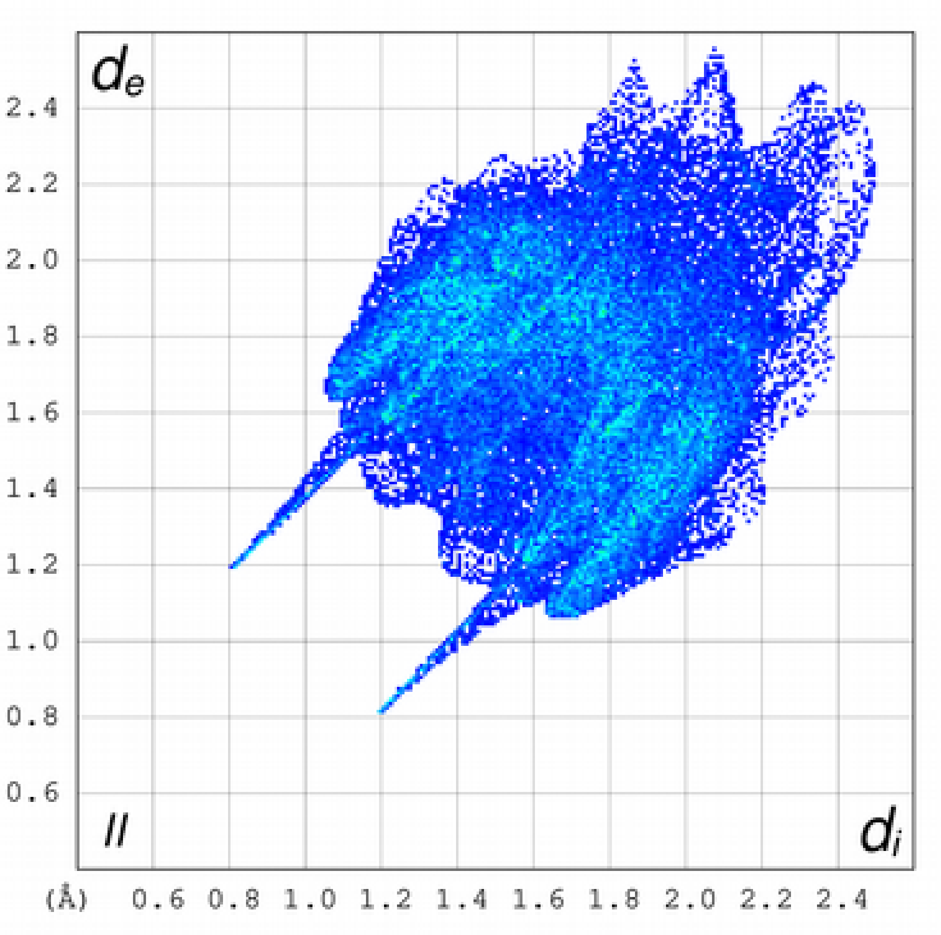

The full two-dimensional fingerprint plot for (II) showing all interactions.
